# Discoid Labral Meniscus Covering Two‐Thirds of a Type C Glenoid: A Case Report

**DOI:** 10.1111/os.12816

**Published:** 2020-12-09

**Authors:** Olof Wolf, Carl Ekholm

**Affiliations:** ^1^ Department of Surgical Sciences, Orthopaedics Uppsala University Uppsala Sweden; ^2^ Institute of Clinical Sciences, Sahlgrenska Academy University of Gothenburg Gothenburg Sweden

**Keywords:** Discoid meniscus‐like labrum, Glenoid dysplasia, Proximal humeral fracture

## Abstract

**Background:**

Glenoid morphology and dysplasia have been extensively described in conjunction with shoulder arthritis. Dysplastic glenoids have a substantial inherent retroversion, a deficient posteroinferior rim, a short scapular neck, and an inferior inclination of the joint surface. The effect of dysplasia on fracture surgery has not been reported to the same extent.

**Case presentation:**

A 65‐year‐old man presented with a proximal humeral fracture. The patient was scheduled for osteosynthesis. The head was deemed unrepairable at the time of surgery and the operative plan changed to replace the proximal humerus. A discoid meniscus‐like labral extension covering two‐thirds of the glenoid was encountered. This finding covered a dysplastic glenoid. The combination of a fracture and a dysplastic glenoid had not been accounted for and made the reconstruction more difficult. The patient received a reverse total shoulder arthroplasty after perioperative considerations regarding reconstruction. At the 2‐month follow up, the patient had a satisfactory clinical outcome, with 90° of flexion and minimal residual pain.

**Conclusion:**

This case illustrates that elective disorders with dysplasia also present to the fracture team. Careful analysis of preoperative imaging should result in an operative plan taking unexpected findings into account.

## Graphical abstract

Three‐dimensional CT‐scan of four‐part proximal humeral fracture in a patient with dysplastic Walch type C glenoid. From this view, the typical glenoid dent and absent scapular neck are visible.

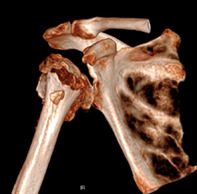

## Introduction

Glenoid morphology has been comprehensively studied and the Walch classification system describes the different glenoids typically observed when treating arthritic shoulders^1^. These arthritic glenoids show an increasing amount of wear and change of appearance. Retroversion is gradually affected and sometimes a biconcave joint surface develops with posterior subluxation of the humeral head (B2 glenoid). Dysplastic glenoids, however, can have the same appearance as the type B3 glenoid with a severe retroversion and a uniconcave joint. Furthermore, the glenoid neck of the scapula and posteroinferior rim show bone deficiency in dysplastic type C glenoids^2^, ^3^.

We describe a 65‐year‐old man who sustained a multifragmentary proximal humeral fracture from a simple fall. A preoperative CT scan revealed a dent in the glenoid cavity that was interpreted to be caused by the fracture. During surgery, an unexpected abnormal glenoid appearance was encountered. A discoid meniscus‐like tissue attached to the labrum covered the lower two‐thirds of the glenoid.

## Case Report

### *History and Preoperative Work Up*

**Fig. 1:**
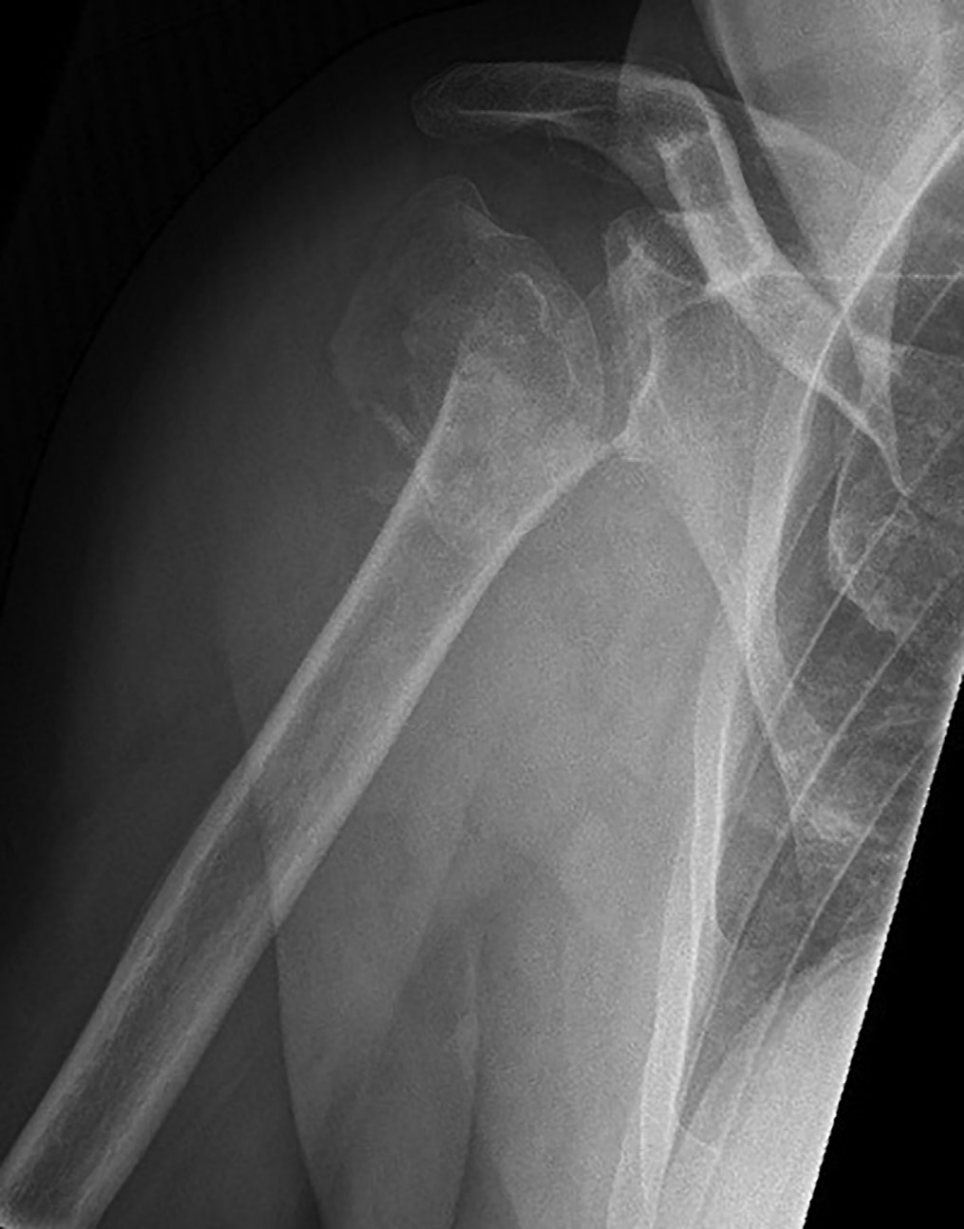
Radiograph from our emergency department displaying a three–four‐part fracture. The humeral shaft is medialized. The dent in the glenoid is believed to be caused by trauma. There is a potential head split component because of two overlapping circular components.

**Fig. 2:**
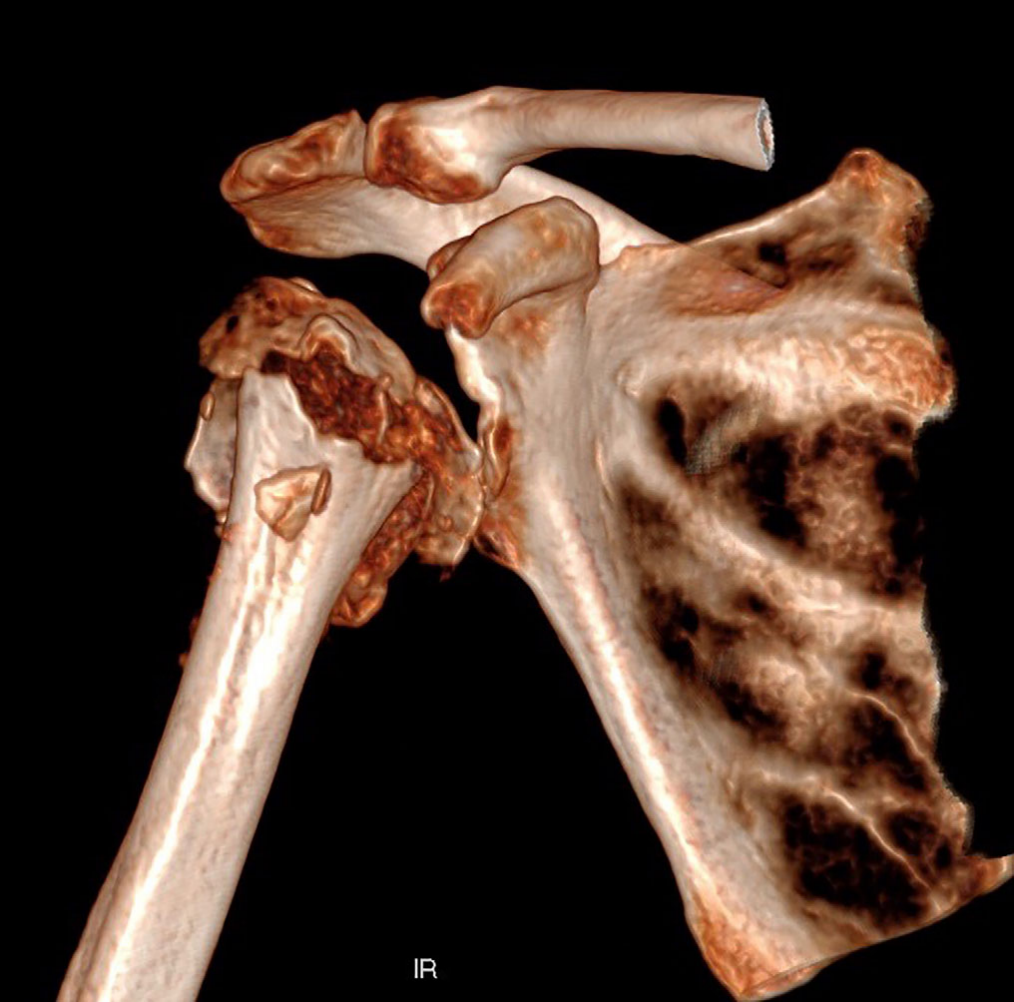
Three‐dimensional CT image displaying a four‐part fracture with humeral head displacement from the shaft. The dent in the glenoid is visible. Head split component with small medial head piece in varus configuration.

**Fig. 3:**
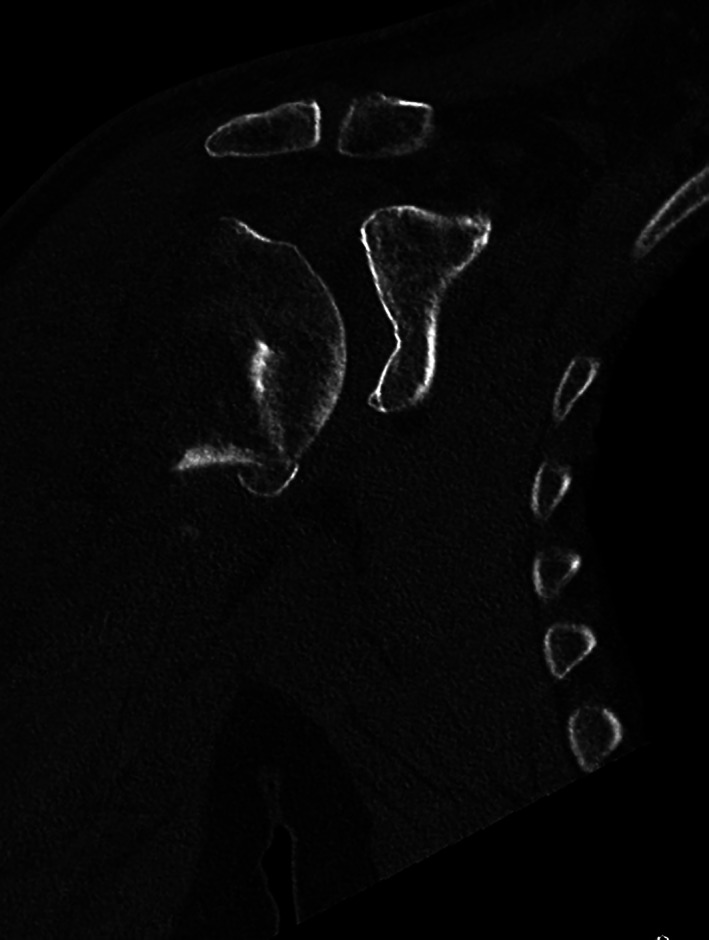
CT image depicting a glenoid dent often associated with dysplastic glenoids.

A 65‐year‐old man sustained a proximal humeral fracture in a previously healthy shoulder. X‐ray examination upon arrival at the emergency department showed a three–four‐part fracture (Fig. 1). He was treated with a sling and scheduled for a CT scan in the outpatient fracture clinic. The scan revealed a multifragmentary four‐part fracture with the shaft separated from the humeral head (Fig. 2). The registrar at the fracture clinic scheduled the patient for surgery, informing him of possible shoulder replacement arthroplasty. The presence of an indentation was also observed in the lower part of the glenoid, believed to be caused by the trauma (Fig. 3).

### *Surgical Procedure*

**Fig. 4:**
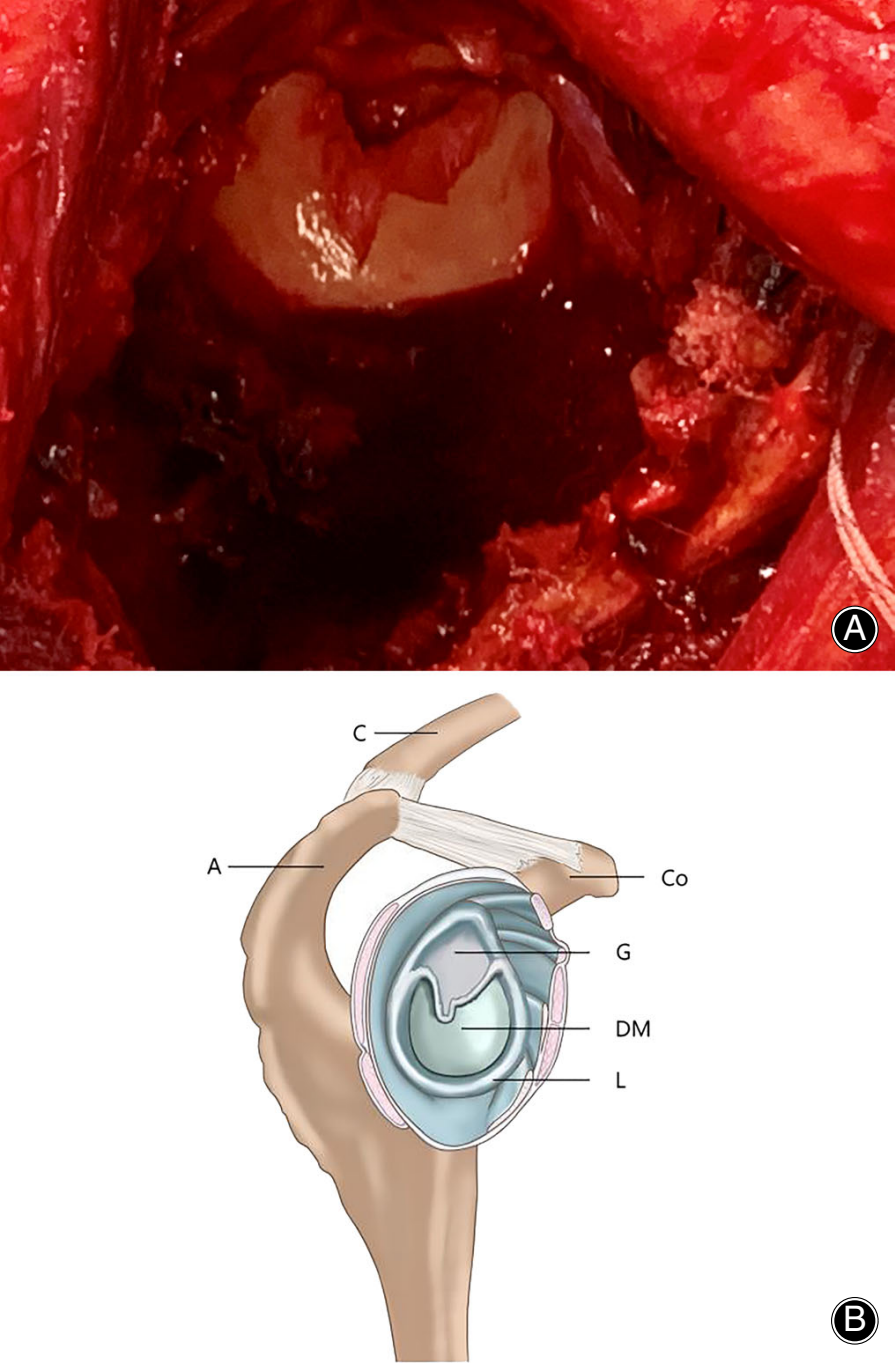
(A) Discoid meniscus covering two‐thirds of the inferior glenoid in the present case. The meniscoid tissue in continuity with the labrum. (B) Drawing illustrating the discoid meniscus (DM) in continuity with the thickened labrum (L) in relation to the native glenoid surface (G). The coracoid process (Co), acromion (A), and clavicle (C) for anatomic overview.

**Fig. 5:**
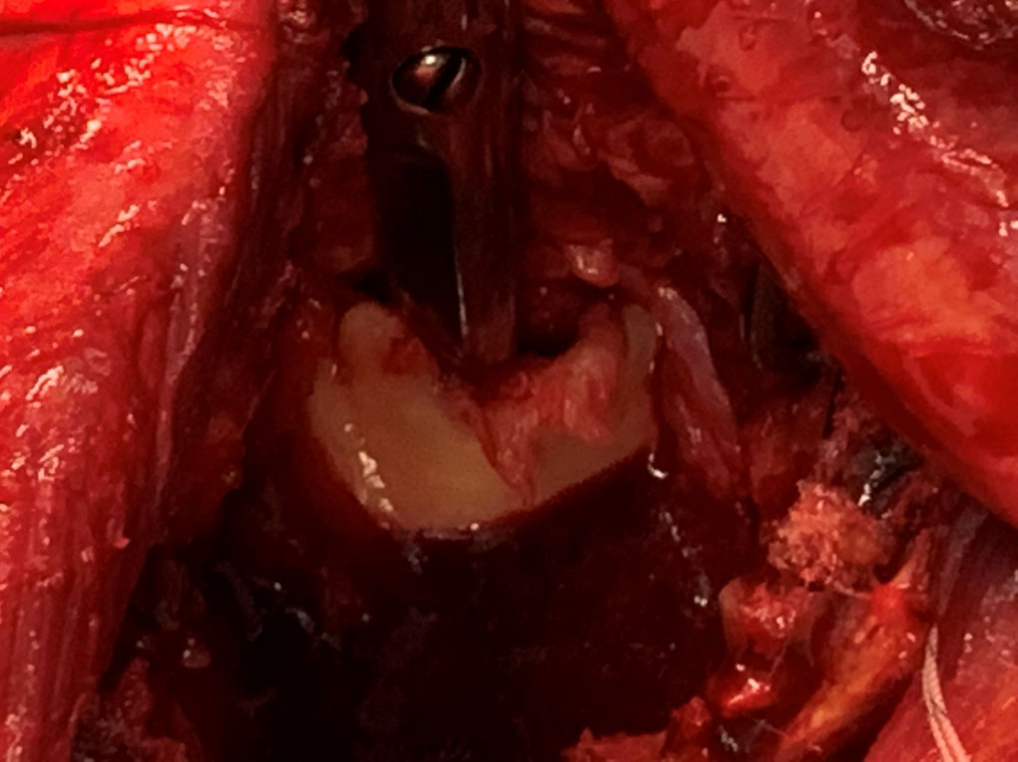
Sub‐meniscoid pocket (a pair of scissors under the meniscoid tissue). Meniscoid tissue attached to labrum except in uncovered cranial part.

The surgical procedure was performed in the beach chair position under antibiotic prophylaxis. General anesthesia was complemented with a brachial plexus block for postoperative pain relief. A standard deltoid splitting approach was used. Once the tuberosities had been tagged and separated, the humeral head showed two horizontal fracture lines and was deemed inappropriate for osteosynthesis. The humeral shaft was found to pierce the subscapularis, resulting in only a fraction of the subscapularis tendon attaching to the lesser tuberosity. After removal of the humeral head, the exposed glenoid was clearly atypical in appearance. The lower two thirds of the glenoid were covered with what was first thought to be a loose layer of cartilage (Fig. 4). However, closer inspection revealed that the thicker periphery of this tissue layer was in continuity with the labrum, but its thinner cranial edge crossing the glenoid surface was not attached to the underlying glenoid surface. Hence, a pocket was formed between the glenoid and the cartilaginous tissue, similar to geniculate discoid meniscus (Fig. 5). In preparing for a reverse total shoulder arthroplasty (RTSA) the “meniscus” was removed, exposing an underlying glenoid that was dysplastic and without cartilage.

After the central guide pin was positioned by the metaglene guide, symmetrical reaming was performed. The central peg exited anteriorly in the scapula, fixing the metaglene component in a slightly retroverted position. Good purchase of the locking screws was achieved, although optimal screw trajectory was difficult to obtain because of the dysplastic nature of the glenoid which had been overlooked preoperatively in the presence of the humeral fracture. Using the standard technique, a Delta Xtend RTSA was implanted, with the tuberosities sutured to the humerus and prosthetic stem.

### *Perioperative and Postoperative Considerations*

**Fig. 6:**
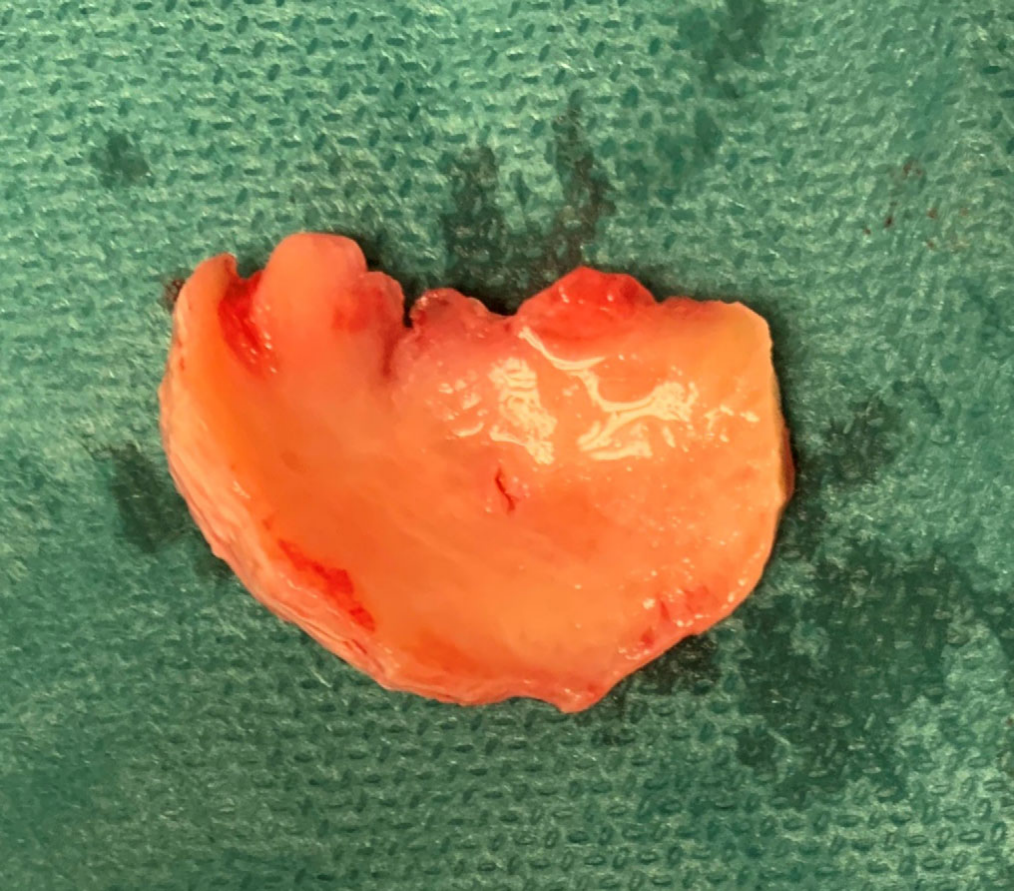
Excised discoid meniscus on side table: approximately 4 × 2.5 cm and 3–4 mm thick, cut from labrum, with cranial part untouched.

**Fig. 7:**
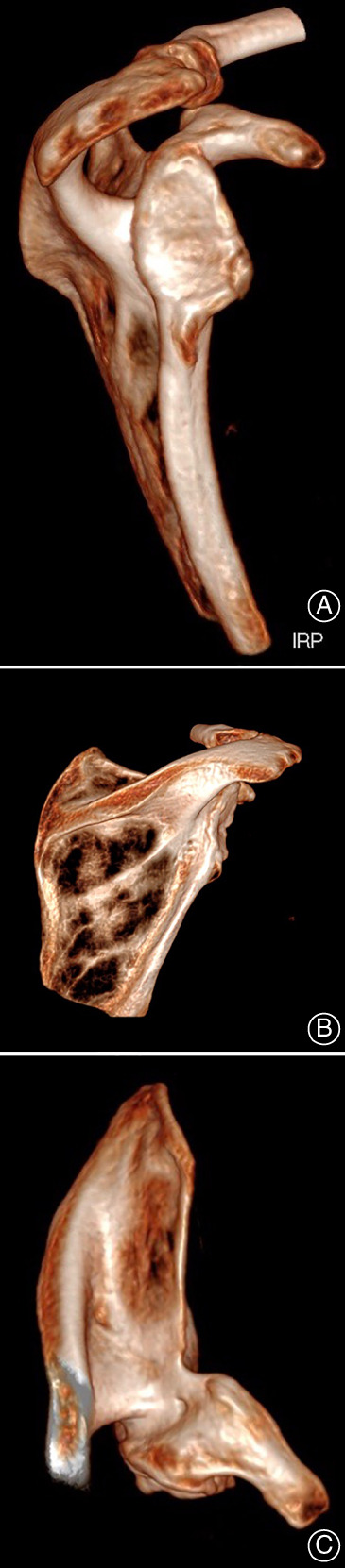
Three‐dimensional CT image of the dysplastic glenoid: (A) lateral (B) posterior, and (C) cranial. The image shows a typical dysplastic glenoid with deficient posteroinferior rim, absence of glenoid neck, retroversion, and inferior inclination of joint surface.

**Fig. 8:**
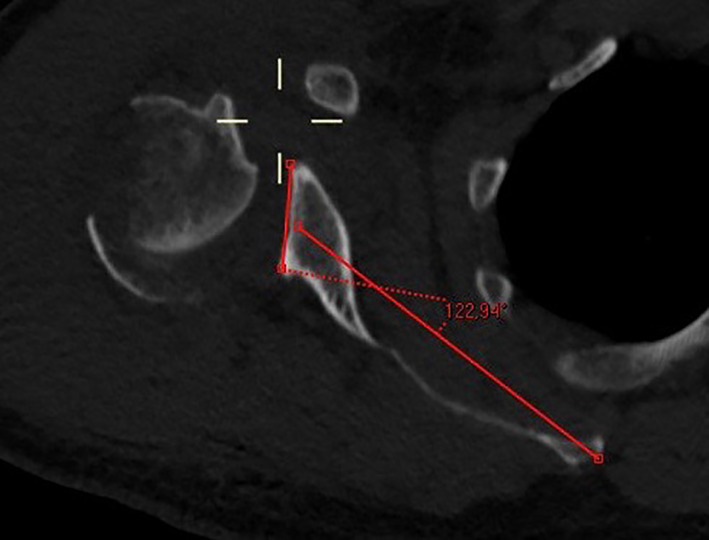
Axial CT slice in the scapular plane displaying glenoid retroversion of >30°, as often seen in dysplastic Walch C type glenoids. The greater tuberosity is detached from the humeral head in this four‐part proximal humeral fracture.

Examination of the excised tissue layer (Fig. 6) revealed a separate layer of tissue without any bony undersurface. The inferior part was approximately 2‐mm thick, gradually becoming thinner towards the cranial edge. This discoid meniscus‐like tissue was only attached to the labrum. The uncovered glenoid was dysplastic, with the absence of a glenoid neck resulting in a steep vertical scapula lateral border (Fig. 7A–C). In the preoperative radiological examination, the glenoid displayed features of dysplasia: absent inferior glenoid neck, 30° inferior inclination of the glenoid surface, and type C glenoid retroversion (Fig. 8).

### *Follow‐Up*

At the 2‐month clinical follow‐up visit, the patient reported almost no pain and satisfactory active assisted range of motion. Active flexion and abduction were just over 90° in the supine position. The patient could reach the top of his head in posterior external rotation and the lumbar spine in posterior internal rotation.

## Discussion

Glenoid morphology has been thoroughly studied and the Walch classification system differentiates concentric from subluxed glenohumeral joints^1^. Type A glenoids are concentric with minimal (A1) or major (A2) wear. In type B glenoids, the humeral head is posteriorly subluxed. B1 glenoids have minimal wear or erosion, whereas B2 glenoids have substantial posteroinferior wear/erosion, resulting in a biconcave joint surface. During the last 5 years, the classification has been modified, adding the uniconcave B3 glenoid with substantial retroversion and joint surface^2^, ^3^. While the B3 glenoid has an acquired retroversion, the type C glenoid is a retroverted uniconcave glenoid caused by dysplasia. The type C glenoid has been characterized as having at least 25° of retroversion, a deficient posteroinferior rim, a short glenoid neck with inferior inclination of the joint surface^4^, ^5^, and central glenoid notching in 28% of cases^6^. This description fits the glenoid of our patient, which was initially overlooked, the focus being on the humeral fracture.

Dysplastic glenoids were previously considered extremely rare: for instance, in a report by Paul *et al*., dysplastic glenoids accounted for only 1.8% of the 1437 shoulder referrals^6^. In contrast, in a group of 98 patients with shoulder symptoms selected for MRI arthrograms, moderate to severe dysplastic glenoids were reported in 14% of cases.^7^ The study also observed posterior labral thickening, replacing the osseous defect of the posterior rim. Failure of ossification of the inferior glenoid ossification center may give rise to labral hypertrophy^8^. In a radiographic study of 16 patients with a hypoplastic glenoid neck, the hypoplastic bone was found to be replaced by fat in 5 patients and fibrocartilage in 6^9^.

In our patient, the labral thickening was not confined to the posterior rim; instead, it stretched across the glenoid from the 9 to the 3 o'clock position, covering the entire inferior glenoid (Fig. 4). The patient was, at the age of 65 years, previously asymptomatic, with full shoulder function despite the pronounced glenoid dysplasia. It seems reasonable that his glenoid bony deficiency was, therefore, well compensated by the labral expansion and that the shoulder mechanics were close to normal.

The outcome of shoulder arthroplasty in patients with dysplastic glenoids is less consistent compared with patients with arthritic wear only. Shoulder hemiarthroplasty produces satisfactory clinical results in most cases^10^, but in other series it has shown a high revision rate due to persistent pain^11^. Total shoulder arthroplasty also tends to produce unsatisfactory outcomes, largely because of glenoid component issues^12^. However, in a recent small series of seven patients with either severe type A2 or type C glenoids, acceptable short‐term functional results and pain relief were achieved using an inlay mini‐glenoid component^13^.

Non‐surgical treatment is possible for many proximal humeral fractures, although in this rather young patient with an unstable fracture configuration, a small humeral head, and no contact between the head and the shaft, this was considered inappropriate. Because our patient was previously asymptomatic and because the part of the glenoid not covered by the labral expansion appeared to have normal cartilage, a standard fracture hemiarthroplasty could have been considered. In our practice we tend to use RTSA for fractures in elderly patients (>70 years). In the present case, with a proximal humeral fracture with the subscapularis pierced by the humeral shaft in combination with the type C glenoid, an RTSA was chosen as the more predictable operative treatment option. At the 2‐month clinical follow‐up, our patient had a good active range of motion and almost no pain.

C type glenoids have been described elsewhere with labral hyperplasia^14^. This discoid meniscus‐like labral extension, covering two‐thirds of the dysplastic glenoid, has not been previously reported. Our case brings new information about dysplastic glenoids. This case also illustrates the need for preoperative analysis for a glenoid component in fracture cases when osteosynthesis is planned, should arthroplasty turn out to be necessary. An RTSA with eccentric glenoid reaming or bone grafting is a viable treatment option.
